# Validation of phylogenetic informative Y-InDels in Y-chromosomal haplogroup O-M175

**DOI:** 10.3389/fgene.2023.1182028

**Published:** 2023-05-02

**Authors:** Zhihan Zhou, Zhimin Li, Yining Yao, Jinglei Qian, Qiqi Ji, Chengchen Shao, Jianhui Xie

**Affiliations:** Department of Forensic Medicine, School of Basic Medical Sciences, Fudan University, Shanghai, China

**Keywords:** human genetics, chromosome Y, InDel, haplogroup, O-M175

## Abstract

The Y-chromosomal haplogroup tree, which consists of a group of Y-chromosomal loci with phylogenetic information, has been widely applied in anthropology, archaeology and population genetics. With the continuous updating of the phylogenetic structure, Y-chromosomal haplogroup tree provides more information for recalling the biogeographical origin of Y chromosomes. Generally, Y-chromosomal insertion-deletion polymorphisms (Y-InDels) are genetically stable as Y-chromosomal single nucleotide polymorphisms (Y-SNPs), and therefore carry mutations that can accumulate over generations. In this study, potential phylogenetic informative Y-InDels were filtered out in haplogroup O-M175, which is dominant in East Asia, based on population data retrieved from the 1000 Genomes Project. A group of 22 phylogenetic informative Y-InDels were identified and then assigned to their corresponding subclades of haplogroup O-M175, which provided a supplement for the update and application of Y-chromosomal markers. Especially, four Y-InDels were introduced to define subclades determined using a single Y-SNP.

## 1 Introduction

The Y-chromosomal variants are widely applied in the fields of human genetic studies by virtue of its paternal inheritance (Jobling and Tyler-Smith, 1995; Hammer and Zegura, 1996; Stone et al., 1996; Kayser, 2003; Wang et al., 2014; Kayser, 2017). Among genetic markers on the Y chromosome, Y-chromosomal single nucleotide polymorphisms (Y-SNPs) are genetically stable and widely distributed, which can reveal the steady transmission of genetic material between ancestors and descendants (Xue et al., 2009). A specific set of Y-SNPs accumulates over generations and gradually forms multiple Y-chromosomal haplogroups as humans migrate and populations expand (Jobling and Tyler-Smith, 2003). On this basis, the first uniformly named phylogenetic Y-chromosomal haplogroup tree was published in 2002 and since then the worldwide Y-SNP types have been divided into 20 main Y-chromosomal haplogroups A to T (Underhill et al., 2001; Consortium, 2002).

With the development of detection technology, more validated phylogenetic informative Y-SNPs are identified, thus more refined phylogenetic structure of the Y-chromosomal haplogroups are constantly consummated (Van Geystelen et al., 2013). Recently, a batch of studies demonstrated more evidence for the update of Y-chromosomal haplogroups, resulting in a renewed understanding of the phylogenetic structure of Y-chromosomal haplogroup C, D, J, K and S (Mondal et al., 2017; Kutanan et al., 2018; Haber et al., 2019; Kanzawa-Kiriyama et al., 2019; Pinotti et al., 2019; Urasin et al., 2019). The continually updated Y-chromosomal haplogroup tree and its subclades provide a refined Y-chromosomal phylogenetic topology structure to further investigate population evolution in more recent era, which facilitates the understanding of the biogeographical origins of ethnic minorities and more specific populations in the application of molecular population anthropology. Besides, the Y-chromosomal haplogroup tree has also attracted the attention of forensic scientists. Previous studies have reported similarity or consistency in unrelated male individuals and father-son discordance during Y-chromosomal STR analysis (Jung et al., 2016; Wang et al., 2016; Zhou et al., 2018). While current Y-chromosomal STR typing cannot make a definite exclusion, the information of Y-chromosomal haplogroups/sub-haplogroups can provide a useful supplement.

Y-chromosomal insertion-deletion polymorphisms (Y-InDels) were generated during the transmission between father and son with stable inheritance (Nachman and Crowell, 2000). As Y-SNPs, such mutations have been passed down along with human evolution and migration, allowing them to be used to trace biogeographical origins. Currently, little concern was paid to phylogenetic information carried by Y-InDels. In Y-chromosomal haplogroup tree 2019–2020, Y-chromosomal haplogroups/sub-haplogroups were mainly defined using Y-SNPs, while Y-InDels accounted for only a very small fraction. In fact, Y-InDels with fragment length polymorphisms confer to convenience in practice (Mills et al., 2006).

The Y-chromosomal haplogroup O-M175 is overwhelmingly dominant in East Asia (Jin and Su, 2000; Karafet et al., 2001; Karafet et al., 2010; Zhong et al., 2011). Recent surveys have shown that haplogroup O-M175 and its sub-haplogroups represent a high proportion in the Chinese population, reaching more than 80% (Lang et al., 2019; Song et al., 2019; Zhang et al., 2020; Wang et al., 2022; Tao et al., 2023). The dominance of the haplogroup O-M175 has brought much focus on its refined phylogenetic structure. Previous researches have investigated potential phylogenetic informative Y-SNPs and provided evidences of newly topologies of haplogroup O-M175 (Yan et al., 2011; Naitoh et al., 2013; Kwon et al., 2015; Ning et al., 2016).

In the current study, we aimed to identify a subset of phylogenetic informative Y-InDels in haplogroup O-M175. After selection of potential Y-InDels, the validation experiments were carried out. Moreover, we reviewed the topology structure of haplogroup O-M175, then assigned candidate Y-InDels to corresponding subclades.

## 2 Materials and methods

### 2.1 DNA sample preparation

Blood samples were collected from unrelated males in a Chinese Han population with written informed consent according to protocols approved by the Ethics Committee of the School of Basic Medical Sciences, Fudan University. DNA extractions were performed according to Chelex-100 procedure (Walsh et al., 1991).

### 2.2 Selection of potential phylogenetic informative Y-InDels in haplogroup O-M175

To select potential phylogenetic informative Y-InDels in haplogroup O-M175, the variant call format (VCF) file from the 30× sequencing data of the fourth phase 1,000 Genomes Project were analyzed (Byrska-Bishop et al., 2022). All male individuals were assigned to the corresponding Y-haplogroups using yhaplo package (Poznik, 2016) based on Python 3.11.1 (https://www.python.org/), with an index manually updated according to the Y-chromosomal haplogroup tree 2019–2020. After obtaining the data of all sequenced bi-allelic Y-InDels, the allele frequencies of each Y-InDel in each Y-chromosomal haplogroup/sub-haplogroup were calculated by direct counting. The Y-InDels which were polymorphic only in haplogroup O-M175 were pending further screening.

With frequencies of candidate Y-InDels in each subclade of haplogroup O-M175, target Y-InDels were screened following two criteria: first, the candidate Y-InDel was mutant in all individuals of the corresponding sub-haplogroup and its subclade; second, the candidate Y-InDel was not mutated in all individuals except the corresponding sub-haplogroup and its subclade. The first principle ensures that the mutation status of candidate Y-InDel does not result from random mutations, and the second principle guarantees the specificity of the candidate Y-InDel.

### 2.3 Primer design, PCR amplification and capillary electrophoresis

Primer pairs were designed using the Primer Premier 5 software (PREMIER Biosoft, CA, United States), considering following criteria: amplicon size limited between 200 bp to 1,000 bp; Tm values from 55°C to 65°C; GC content from 40% to 60%; avoiding primer-dimer, hairpin loop formations and complementary 3′-ends. The specificity of DNA sequences and primer pairs was evaluated using the BLAST-Like Alignment Tool (Kent, 2002) and the Basic Local Alignment Search Tool (Altschul et al., 1990), respectively. For capillary electrophoresis analysis, one of each primer sets were modified with fluorescent dyes (FAM or HEX) at the 5′-ends. All primer sets were validated by agarose gel electrophoresis.

PCR amplification was performed in a volume of 30 μL, consisted of 15 μL of 2× Taq Master Mix (Novoprotein, China), 0.3 μL of primer and 1.5 μL of template DNA. Thermal cycling was performed in a GeneAmp PCR System 9,700 (Applied Biosystems, CA, United States), with an initial denaturation step at 94°C for 90 s, followed by 35 cycles of 94°C for 30 s, 58°C for 60 s, 72°C for 60 s, then a final extension at 72°C for 10 min, and maintained at 4°C.

To detect the genotype of the candidate Y-InDels, the PCR products were first subjected to capillary electrophoresis. The samples were prepared by combination of 1 μL PCR products and 9 μL of a 19:1 mixture of deionized Hi-Di™ Formamide (Thermo Fisher Scientific, MA, United States) and GeneScan™ 500 LIZ^®^ size standard (Thermo Fisher Scientific, MA, United States). The mixture was denatured at 95°C for 5 min and then chilled at 4°C. The products were separated by ABI PRISM 3130xL Genetic Analyzer (Applied Biosystems, CA, United States). The run data were analyzed using GeneMapper^®^ ID software v3.2 (Applied Biosystems, CA, United States). Genomic DNA 2800M (Promega, United States) was applied to be a positive control.

### 2.4 Validation of potential phylogenetic informative Y-InDels

To validate potential phylogenetic informative Y-InDels, male samples belonging to the haplogroup O-M175 were screened out by a previously developed 16-plex Y-SNP typing system (Zhou et al., 2020) for subsequent experiments. With genotype of candidate Y-InDels, a series of Sanger sequencing were performed (Saiheng Biotechnology Co., Shanghai, China). Both wild-type and mutant samples of each candidate Y-InDel were sequenced to confirm the alleles of loci. After that, we selected the representative Y-SNPs of the corresponding subclades of each candidate Y-InDel. Next, we sequenced those representative Y-SNPs for wild-type and mutant samples. After obtaining the sequencing results, the mutation status of the candidate Y-InDels was compared with that of the subclade-determining Y-SNPs to determine the subclades which the candidate Y-InDels belonged to. In addition, we cross-validated the haplogroup/sub-haplogroup assignment of newly identified Y-InDels and the 16-plex Y-SNP typing system (Zhou et al., 2020) using 235 male samples.

## 3 Results

### 3.1 Selection of potential phylogenetic informative Y-InDels by data retrieved from 1,000 Genomes Project

After initial processing of sequencing data from 1000 Genomes Project, a total of 23,726 bi-allelic Y-InDels on 1233 Y chromosomes were acquired. After the frequencies of Y-InDels in each Y-chromosomal haplogroup was calculated, a batch of 2,375 Y-InDels were variant only in haplogroup O-M175. Following screening criteria, a number of 614 potential phylogenetic informative Y-InDels and their corresponding subclades were identified. This batch of Y-InDels and the representative Y-InDels of the Y-chromosomal tree 2019–2020 were compared to remove identical Y-InDels, namely, M175, M111, A15721, M121, FGC12511, M134, M117, M133 and M333. A total of 605 Y-InDels were selected for further screening. It is worth noting that a number of candidate Y-InDels were also eliminated due to their high homology with sequences in other regions or difficulties in their detection. Eventually, a group of 22 Y-InDels was identified as potential phylogenetic informative Y-InDels for subsequent validation. The general information of newly phylogenetic informative Y-InDels is presented in Table 1.

**TABLE 1:** The summary of phylogenetic informative Y-InDels and corresponding subclades in haplogroup O-M175.

Subclades of Y-haplogroup O-M175	Newly identified phylogenetic informative Y-InDels (#rs)	Position (GRCh38)	Mutation information	Number of tested samples
O1a1	rs79011057	15,870,505	C > −	8
O1a1	rs75465866	14,101,642	T > −	8
O1a1a	rs756897195	14,444,770	TTG > −	5
O1a1a1	rs776778598	19,623,371	GAA > −	3
O1b	rs200942940	12,416,056	C > −	8
O1b1	rs760314663	20,774,821	C > −	6
O1b1a	rs749761428	6,796,839	- > T	5
O1b1a1a	rs774008684	17,452,553	AAGA > −	3
O1b1a1a1a1a1	rs774805227	16,596,493	A > −	1
O1b2	rs201451931	7,765,543	T > −	4
O1b2a1a	rs200704310	12,173,532	TAA > −	3
O2a	rs201101541	16,693,737	- > C	10
O2a1a1	rs2044026501	16,599,674	A > −	2
O2a1b	rs1569514296	13,477,987	TAC > −	6
O2a1b1a	rs754348496	12,420,208	T > −	4
O2a1b1a1a1a1	rs759556853	12,663,077	T > −	3
O2a2a	rs768760512	13,353,588	- > T	8
O2a2a1a	rs776330196	13,497,196	T > −	6
O2a2b	rs79480324	14,153,309	A > −	7
O2a2b1a1a1a4a	rs796937681	12,821,143	- > A	2
O2a2b1a2a	rs201510546	8,044,404	TAAAG > −	5
O2a2b1a2a1a	rs762474604	15,235,863	AT > −	2

### 3.2 Identification of phylogenetic information and assignment of candidate Y-InDels to subclades in haplogroup O-M175

The ancestral and derived status of each candidate Y-InDel was first validated using samples. The results from capillary electrophoresis analysis showed the presence of ancestral and derived status of each candidate Y-InDel, which confirms the diversity of candidate Y-InDels in the population. Variants at rs79011057 and rs75465866 are most likely synchronized, as suggested in both data screening and validation experiments.

**FIGURE 1:**
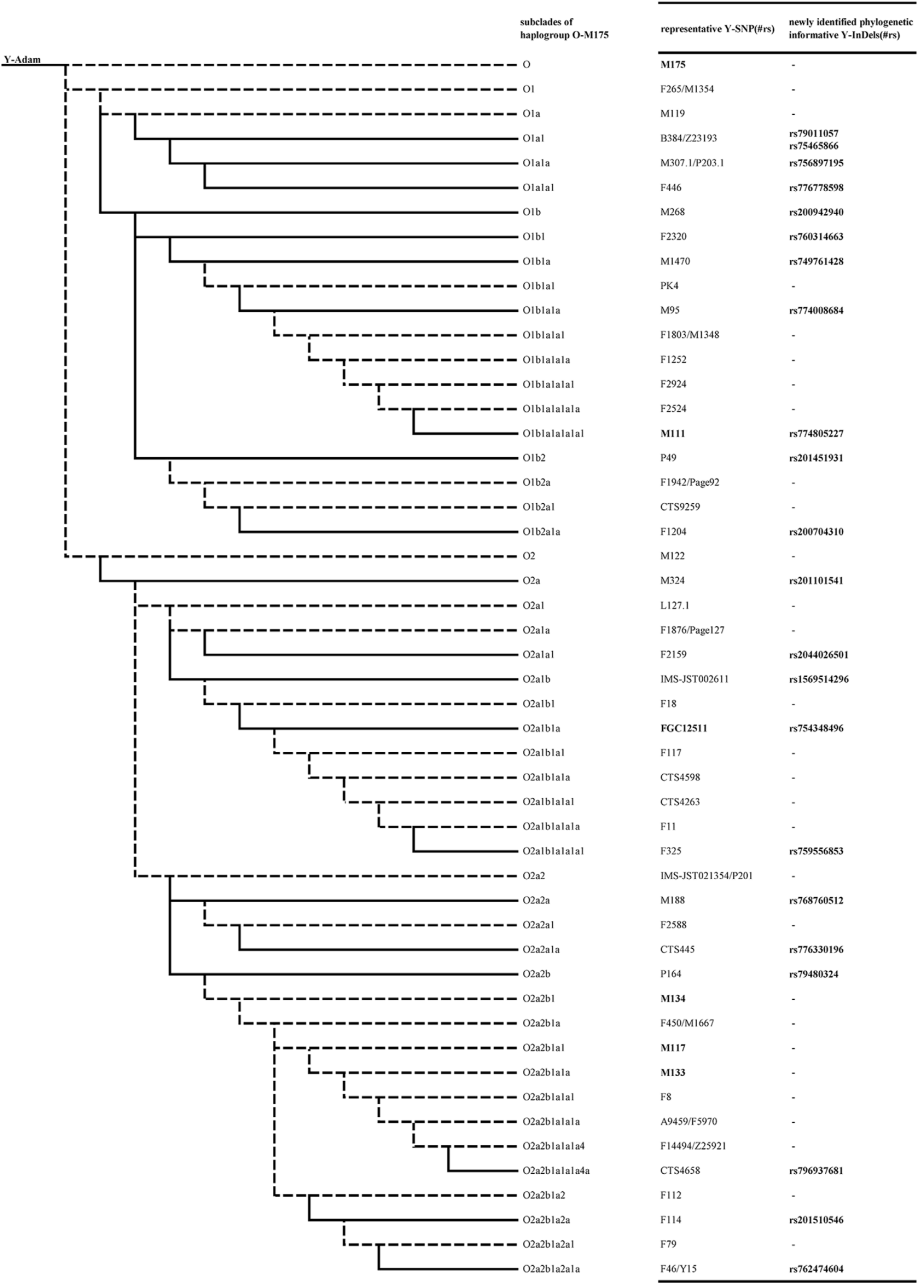
A phylogenetic tree constructed by phylogenetic informative Y-InDels with representative Y-SNPs of corresponding subclades. The dotted lines indicate haplogroups/sub-haplogroups which is not included in the current study. The bold fonts indicate Y-InDel loci.

In order to assign the subclade of candidate Y-InDels, corresponding representative Y-SNPs were sequenced in individuals that may belong to the corresponding subclade. The selected representative Y-SNPs and their corresponding subclades is visually displayed in Figure 1. In each subclade, the mutation status of candidate Y-InDels was validated by sequencing to be consistent with representative Y-SNPs and occurred synchronously in the same samples (Table 1). Thereby, candidate Y-InDels could be assigned to the corresponding subclade of haplogroup O-M175 while tested samples were assigned into corresponding haplogroups/sub-haplogroups. Importantly, five Y-InDels (rs79011057, rs75465866, rs749761428, rs754348496 and rs768760512) could be assigned, respectively, to haplogroup O1a1, O1b1a, O2a1b1a and O2a2a which were defined only using a single Y-SNP in Y-haplogroup tree 2019–2020.

In attempt to provide additional evidence, we performed a previously developed 16-plex system on investigated samples (Zhou et al., 2020). The assignment of Y-haplogroups/sub-haplogroups from the 16-plex system and the newly identified Y-InDels were compared. Although the two sets of loci did not cover the identical Y-chromosomal haplogroups/sub-haplogroups, there were no conflicting assignment in all tested samples. The 16-plex system contains only the main subclades of the haplogroup O-M175, whereas the 22 newly identified Y-InDels can further assign samples to further downstream sub-haplogroups. For example, the 16-plex system contains two subclades, O1b and O1b1a2, and lacks representative sites of other downstream branches of haplogroup O1b. The addition of newly identified Y-InDels could identify haplogroup O1b1 (rs760314663), O1b1a (rs749761428), O1b1a1a (rs774008684), O1b1a1a1a1a1 (rs774805227), O1b2 (rs201451931) and O1b2a1a (rs200704310) (Figure 1), thus providing more detailed Y-chromosomal haplogroup typing results. Furthermore, these 6 Y-InDels showed ancestral status in samples with haplogroup O1b1a2.

## 4 Discussion

In the current study, a number of candidate phylogenetic informative Y-InDels in haplogroup O-M175 were screened by data analysis. Phylogenetic information of candidate Y-InDels was identified by a series of validation experiments, then distributed to the corresponding subclades of haplogroup O-M175. The development of Y-InDel markers can complement the phylogenetic informative markers to enrich the definition and to provide the typing of Y-haplogroup/sub-haplogroup in a convenient manner. In fact, it is difficult to identify new subclades using Y-InDels due to the wide investigation in the definition of subclades of Y-haplogroups using Y-SNPs. However, as a type of genetically stable markers with length polymorphism, Y-InDels has the advantage of convenient detection, which introduces a prospect for its further application.

In this study, the characteristics and advantages of Y-InDels were fully considered, so as to use them as Y-chromosomal phylogenetic informative markers. In haplogroup O-M175 from the Y-chromosomal haplogroup tree 2019–2020, a total of 9 haplogroup/sub-haplogroups can be determined using Y-InDels including haplogroup O-M175, O1b1a1a1a1a1-M111, O1b1a1a1a1a1a1a1a-A15721, O2a1a1a1a1-M121, O2a2b1-M134, O2a1b1a-FGC12511, O2a2b1a1-M117, O2a2b1a1a-M133 and O2a4-M333. The newly phylogenetic informative Y-InDels provided candidates in haplogroup O-M175, especially for the subclades with only one representative Y-SNP in Y-haplogroup tree 2019–2020, such as haplogroup O1a1-B384/Z23193, O1b1a-M1470, O2a1b1a-FGC12511, and O2a2a-M188. The newly phylogenetic informative Y-InDels involved in subclades of several levels. For instance, the rs796937681 corresponding to the 12th level subclade O2a2b1a1a1a4a-CTS4658 of haplogroup O-M175, indicating that the newly phylogenetic informative Y-InDels had the ability to subdivide the haplogroup O-M175. The detailed division of Y-haplogroups can provide more information of biogeographical origins, thus making phylogenetic informative Y-InDels a practical supplement in various researches.

The information of paternal biogeographical origin contained in Y-haplogroup tree has always been the focus of anthropological and archaeological research to trace the history of human evolution and expansion. When the emergence of human out of Africa was confirmed by Y-haplogroups (Underhill et al., 2000), the biogeographical origins of populations have been revealed, with the continuous refinement and renewal of Y-haplogroups/sub-haplogroups (Lall et al., 2021; Platt et al., 2021). Using the newly identified phylogenetic informative Y-InDels from this study, the subdivision of Y-haplogroup targeting the haplogroup O-M175 can be performed in a more convenient form. On the other hand, smaller amplicon size at Y-InDels also facilitate the detection of archaeological samples and ancient DNA. The subdivision of Y-haplogroups provides the possibility for further investigation of population evolution in more recent era, which may improve the understanding of the biogeographical origins of ethnic minorities and more specific populations.

## Data Availability

The raw data supporting the conclusion of this article will be made available by the authors, without undue reservation.
